# Suicidal Thoughts and Behaviours Among Autistic Adults Presenting to the Psychiatric Emergency Department: An Exploratory Chart Review

**DOI:** 10.1007/s10803-021-05102-9

**Published:** 2021-06-14

**Authors:** Patrick Jachyra, Meng-Chuan Lai, Juveria Zaheer, Natasha Fernandes, Michelle Dale, Amanda Sawyer, Yona Lunsky

**Affiliations:** 1 Azrieli Adult Neurodevelopmental Centre, Campbell Family Mental Health Research Institute, Centre for Addiction and Mental Health, Toronto, ON Canada; 2 The Margaret and Wallace McCain Centre for Child, Youth & Family Mental Health, Campbell Family Mental Health Research Institute, Centre for Addiction and Mental Health, Toronto, ON Canada; 3 Department of Psychiatry, The Hospital for Sick Children, Toronto, ON Canada; 4 Department of Psychiatry, Temerty Faculty of Medicine, University of Toronto, Toronto, ON Canada; 5 Autism Research Centre, Department of Psychiatry, University of Cambridge, Cambridge, UK; 6 Department of Psychiatry, National Taiwan University Hospital and College of Medicine, Taipei, Taiwan; 7 Institute for Mental Health Policy Research, Campbell Family Mental Health Research Institute, Centre for Addiction and Mental Health, Toronto, ON Canada

**Keywords:** Suicide, Emergency department, Chart review, Autism, Adult, Life transition, Interpersonal conflicts, Rumination

## Abstract

Despite increasing attention on suicidality in autistic people, we know little about suicidal presentations when autistic individuals present to hospital emergency departments (ED). We conducted an exploratory retrospective chart review of suicidal thoughts and behaviours (STB) of autistic adults who presented to a psychiatric ED. The analysis included 16 charts over a 10-week period. Findings highlight that reported STB were not always the presenting issue. Life transitions and interpersonal conflicts were common antecedents, and active rumination about STB was distressing and fatiguing. Findings imply that ED visits serve as important opportunities for suicidal risk reduction for autistic individuals, through implementation of strategies for identification of STB such as active screening, and the provision of suicide resources tailored to autistic people.

## Introduction

Emerging research suggests that autistic people^1^ think about and engage in suicidal thoughts and behaviours (STB) (e.g., suicide attempts, non-suicidal self-injury) at high rates. A recent systematic review of nine studies reported that rates of suicidal ideation ranged between 11 and 66% in autistic adults (Hedley & Uljaveric, 2018). High rates of suicidal ideation were also noted in a study conducted in the United Kingdom where rates of suicidal ideation in a cohort of autistic adults was nine times higher than what was reported in the general population (Cassidy et al., 2014). Concurrent with high rates of suicidal ideation among autistic people, higher rates of suicide attempts have also been noted where the prevalence of suicide attempts was between 7 to 47% in a systematic review of 12 studies (Zahid & Upthegrove, 2017). Although there is wide variation in the rates of suicidal ideation and death by suicide attempts reported in research, the magnitude of the issue however is also reflected in population-level research where autistic adolescents and young adults in Taiwan had a higher incidence of suicide attempts compared to non-autistic people (Chen et al., 2017). Higher rates of STB observed among autistic people are problematic as registry-based studies in Sweden (Hirvikoski et al., 2020), Denmark (Kõlves et al., 2021), Australia (Hwang et al., 2019) and the United States (Kirby et al., 2019) suggest that autistic people are at increased risks of death by suicide than non-autistic people. Such patterns seem to be more apparent in autistic females, and autistic individuals without intellectual disability (Hirvikoski et al., 2016, 2020; Kirby et al., 2019; Kõlves et al., 2021). Given the increased risks to die by suicide among autistic people, it is clear that STB is a grave concern in need of research to advance the identification, prevention, and intervention of STB.

To date, research examining STB has predominantly drawn on administrative health data and retrospective self-reports. While population-level approaches are valuable in raising awareness of suicide risks amongst autistic people, they do not capture contextual details of what it is like to experience STB. To address this issue, retrospective self-reports of autistic people’s experiences of STB have been studied (Richards et al., 2019). However, retrospective accounts rely on the aspects the person can remember, and may not accurately capture all details associated with STB at the height of a crisis. Despite the strengths of population-level and self-report approaches, there has been a paucity of research that examines what happens when autistic people seek help whilst experiencing STB. To gain further insights, a chart review may be a beneficial tool to examine contextual characteristics and experiences of STB *at the height of a crisis*. Chart reviews have the potential to explicate critical STB information which would not be captured through either administrative or self-report approaches. Another strength of chart reviews is that they can highlight how autistic people are perceived, and how they receive care in a healthcare setting, as what is documented in the chart is a social construction of the relationship between the patient and attending healthcare professionals (HCP). Despite the potential utility of chart reviews, only one chart review study of suicidality based on outpatient charts with autistic children and adolescents (see Karakoç Demirkaya et al., 2016) has been published. To this end, there is no published research that has examined ED charts for STB, and no previous chart reviews conducted with regard to autistic adults.

Given the lack of research using ED charts among autistic adults, little is known about real-time descriptions of STB, the presentations, and the contextual factors or antecedents that accompany STB among autistic people. We therefore conducted a qualitative chart-review to describe and analyze the presentations and clinical encounters of a group of autistic adults experiencing STB who presented to a psychiatric emergency department (ED) in a metropolitan area.

## Study Design and Method

A retrospective chart review of visits to a tertiary psychiatric ED in Toronto, Canada was conducted. This study draws on a larger retrospective chart review which examined all visits made to that ED during a 10-week period (October 2016-Janurary 2017). During this time, 73 individuals with a diagnosis of intellectual or developmental disability visited the ED (See Fernandes et al., 2020). For the purposes of the current study, each of these ED charts were reviewed again, and included for analysis if individuals met the following criteria: (1) having a clinical diagnosis of autism, defined by DSM-5 Autism Spectrum Disorder or equivalent (e.g., Autistic Disorder, Asperger’s Disorder, Pervasive Developmental Disorder Not Otherwise Specified); and (2) if there was any mention of STB at the initial ED visit, or any mention of STB in the hospital medical chart from previous visits, up to four weeks prior to the ED visit.

### Analysis

A qualitative, thematic analysis of the charts was conducted. Informed by Braun & Clarke, (2006) steps of thematic analysis, three-team members commenced analysis by reading each chart to gain an understanding of the presentations, interactions, and outcomes across the charts. Following the reading of each chart, they met to discuss their impressions of the visit to the ED, presentations, and interactions noted in the charts as a way to summarize the study sample. Next, each chart was read again by the primary author, and inductive coding (i.e., assigning a short name to segments of data present in the charts) was used to extract, reduce, and organize the data. Codes that were generated were then grouped into similar categories. The categories were reviewed at a larger team meeting in which the patterns, ideas, and relationships observed across the categories were discussed, debated, and refined. The larger team meeting was integral to the interpretation of the data and enhanced the rigour of the analysis as the research team possessed diverse expertise. The research team consisted of two emergency room psychiatrists working at this particular ED, an expert in suicidology, a child psychiatrist, a clinical psychologist, an occupational therapist, and a post-doctoral researcher training in suicidality among autistic youth and adults.

After establishing the categories described above, analysis continued by conducting a cross-comparison of data from each participant, and analysis across each category was completed to draw out patterns and relationships observed in each category (Jachyra et al., 2015). Data generated during the cross-analysis was used to examine recurring patterns across the dataset. When conducting this cross-analysis, memos were written as a tool to help draw out relationships observed across the categories, and negative cases (i.e., similar to outliers in quantitative research) which contradicted preliminary findings were included to examine nuances in the data and explore alternative directions. To enhance the rigour of the interpretations, negative cases were used to test the rigour of the study findings (Jachyra et al., 2021). Analysis continued by grouping and collapsing individual categories into three themes: Presentations of STB, Contextual Factors, and Clinical Dispositions. These themes captured the patterns and relationships that were most commonly observed across the charts, and identified as primary themes. Analysis was considered complete when these three primary themes were established, and agreed to by all team members during a final group analysis meeting.

## Results

### Demographics

**Fig. 1:**
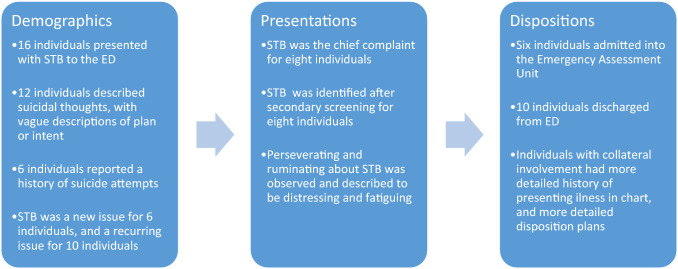
Summary of Study Findings

In total, 24 individuals had a diagnosis of autism and presented to the ED, and among them 16 individuals presented with STB. Individuals ranged from 17 to 31 years of age (mean 22.5 years). There were 10 males and 6 females. The majority of individuals (75%) described suicidal thoughts, with vague descriptions of plans and intent. Six ﻿individuals had a reported history of suicide attempts (37%), and 5 had a history of previous self-harm (31%). STB was a new issue for 6 individuals (37) and was a recurring issue for 10 individuals (62%). Tables 1 and 2 summarize the demographic profiles, and Fig. 1 provides an overview of the study findings.

**Table 1:** Demographic information

Age	Self-identified Gender	Self-identified Ethnicity	Education level	Employment Status	Housing Arrangement	Co-occurring diagnoses
≤ 20	Male	White	Some high school	Family support	Living with roommates	Rule out SSD
21–25	Male	Asian	High school completed and some post-secondary	DSP	Living with family	MDD, GAD, SSD
21–25	Male	N/A	High school and post-secondary completed	DSP	Living in group home	MDD
21–25	Female	Korean	High school completed and some post-secondary	DSP	Living with family	N/A
≤ 20	Female	White	Attending post-secondary education	Student	Living with family	BPD
21–25	Male	Korean	High school completed	Family support	Living with family	OCD, PD
21–25	Male	Jamaican	Elementary school	DSP	Living with family	ADHD
21–25	Male	Jewish	High school completed	Employed	Living with family	GAD
21–25	Male	White	Some high school	DSP	Living with family	ADHD, MDD, SUD
26–30	Female	White	N/A	N/A	Living with roommates	BPD
26–30	Female	White	Some high school	DSP	Living with family	MDD, GAD, PTSD
21–25	Male	White	High school completed	DSP	Living alone	ADHD, GAD, OUD, PTSD
≤ 20	Male	N/A	In high school, but not attending	Employed	Living with roommates	BPD, ASPD
≥ 31	Male	White	Some high school	Employed	Living alone	MDD, AD, OCD
21–25	Female	N/A	High school and post-secondary complete	N/A	Living with family	ADHD, BAD, AN, BN
21–25	Female	N/A	High school completed	Employed	Living with common-law partner	GAD, MDD, PTSD, OCD

*AD* Adjustment Disorder, *ADHD* Attention-Deficit/Hyperactivity Disorder, *ASPD* Anti-Social Personality Disorder, *AN* Anorexia Nervosa, *BAD* Bipolar Affective Disorder, BN Bulimia Nervosa, *BPD* Borderline Personality Disorder, *GAD* Generalized Anxiety Disorder, *MDD* Major Depressive Disorder, *OCD* Obsessive–Compulsive Disorder, *OUD* Opioid Use Disorder, *PD* Panic Disorder, *PTSD* Post-Traumatic Stress Disorder, *SSD* Schizophrenia Spectrum Disorders, *SUD* Substance Use Disorder, *DSP* Disability Support Program, *N*/*A* Information not available

**Table 2:** Presenting issues and disposition

Reason for Attending ED	STB Details	Accompanied to ED with	STB History	History of Attempts	History of self-harm	Collateral involvement	Disposition
STB	SI	Friend	On/off for 2 years	Put a knife to throat and wrist	No	No	Discharged
STB	SI	Alone	No	No	No	Yes	Admitted
STB	SI	Alone (someone suggested to attend)	Onset for past 1.5 years	Cord around neck	No	No	Discharged
Assaulted homeless person	SI	Ambulance transport	No	No	No	No	Admitted
STB	SI	With mother	Yes	Jump out of moving car	No	Yes	Admitted
Substance use	SI	With mother and step-father	Yes	No	No	Yes	Discharged
Requesting medication	SI	Alone	Yes, 7 years	Jump from building, overdose, strangulation	Cutting, punch eyes, nose and face	No	Discharged
Break up with partner	SI	With mother and case worker	No	No	No	Yes	Discharged
Diagnostic clarification	SI	With mother	Yes	No	Overdose, cutting	Yes	Admitted
Cocaine addiction	SI	Alone	Yes	No	No	No	Offered admission but declined
Financial assistance	SI	Alone	Yes	No	Cutting	No	Discharged
Medication review	SI	Alone	No	No	No	Yes	Admitted
STB	SI and planned attempt	Brought in by police	No	No	No	No	Discharged
STB	SI with plans of overdose or cutting	Mother	On/off for past month	Cutting, 3–4 weeks ago	Cutting thigh with razor	Yes	Admitted
STB	SI and began writing suicide note, with plan to jump from bridge	Alone	No	No	No	No	Discharged
STB	SI and plan	Brought in by police	Yes	Yes	Cutting	No	Discharged

*SI* Suicidal ideation, *STB* Suicidal thoughts and behaviour

### Presentations of STB

Upon arrival to the ED, there was variation in how and when STB was identified. For eight individuals (50%), STB was considered to be the presenting issue (i.e., ‘chief complaint’) and was immediately identified by attending HCPs. For example, one individual contemplated jumping from the second level while in a shopping mall and went swimming immediately after in an effort to calm himself down. After self-concerns of drowning while swimming, he presented to the ED at the suggestion of the pool staff. For the other half of the sample, STB was noted during the mental status exam conducted in the ED which included detailed STB screening. These individuals presented to the ED initially seeking support for other issues such as a financial crisis, diagnostic clarification, psychiatric evaluation after assaulting someone, request for medication, and medication review. In one instance, an individual presented to the ED after feeling restless, irritable, and on edge after not taking his psychotropic medications for three days. When asked about STB during the mental status exam, he reported that he thought of jumping off a balcony, thought of taking a photo standing on the ledge of the balcony as a way to ‘practice’ his death, and described he had a hard time letting go of these thoughts as he repeatedly thought of doing it for three days. Although he never took the photo to ‘practice’, his initial presentation in the ED highlights the variability in possible STB presentations in autistic adults.

Despite differences observed in the chief complaint, one commonality was that autistic individuals experienced difficulties managing recurring thoughts about STB. At the time they presented to the ED, 10 individuals (62%) perseverated about suicidal thoughts, plans, or a previous attempt. For example, one individual described she was ‘stuck’ thinking about her attempt to overdose on medication which she described was emotionally unsettling. Another individual expressed to the HCP that he was tired of thinking about suicide as ‘it just does not go away’. As illustrated in the quotes above, perseverating and ruminating about STB not only was debilitating, but also exacerbated an already difficult experience.

### Contextual Factors

Across the charts, STB occurred and were exacerbated by broader life circumstances such as interpersonal conflicts. For 5 individuals (31%), STB was reported in the context of a conflict or misunderstanding. For example, one individual reported that he had strong urges to jump off a bridge for two days, and began writing a note to his family after a recurring conflict with his family. Another individual thought of jumping in front of an underground train, after trying to mediate a conflict with her friends. After walking into the train boarding level, she called the crisis hotline, and was brought to the ED by the police. For another individual, numerous conflicts about adhering to rules, routines and structures after moving back home with his parents were the wider contextual factors that were occurring when he jumped out of a moving vehicle while his parent was driving.

Another contextually relevant factor observed across 31% of the charts was the role of life transitions. STB were reported shortly after transitions occurred such as transitioning into post-secondary education (university/college studies), and transition into a new living arrangement (such as moving into a group home, or moving back home with parents). For three individuals (18%), recent transitions into new scholastic programs were accompanied by difficulties in adapting to the new environment, scholastic demands, and social interactions. For one individual, social pressure of attending networking events that were mandatory in his program of study, combined with changes to his social supports (new living arrangement) after transitioning into post-secondary education triggered the onset of STB. After worsening ideation with thoughts of death by suicide attempt, he presented to the ED with his friend, and he noted to the HCP that his ‘suicidal thoughts have been stronger than in the past, and now effecting my life and the way I interact with others’. While interpersonal conflicts and life transitions were likely only two aspects that may have been contributory or precipitating factors in these contexts, they highlight the importance of situating STB within broader life histories, along with social and contextual forces that are present in the lives of autistic adults.

### Clinical Dispositions

During clinical assessment at the ED, co-occurring mental health conditions (most commonly anxiety and depression) and substance use were commonly identified. 15 individuals (93%) had at least one co-occurring mental health condition noted in their chart. 6 individuals (37%) reported using alcohol, and other substances also were documented which included the use of marijuana (4 individuals), methamphetamines (2 individuals), crack/cocaine (2 individuals), psilocybin mushrooms (1 individual) and heroin (1 individual). Substance use was a compounding factor when assessing STB, as HCPs noted that it was difficult to discriminate whether STB were substance induced, for three individuals. Although substance use was identified as a compounding issue, underlying substance use was not directly addressed during the ED visit. Apart from one individual who received a referral to a detoxification program, dispositions focused on the acute STB issues at hand, with no documentation in the chart that individuals were counselled about substance use/abuse while attending the ED.

Following evaluation, the majority of individuals (62%) were discharged to community care, with a referral or a recommendation to see to a psychiatrist or a psychologist in the community, or a follow-up appointment with a general practitioner. 6 individuals (37%) were temporarily admitted into the hospital Emergency Assessment Unit (EAU) and held in the ED for further assessment. Individuals who were admitted into the EAU had an element of active STB risk determined by the HCP. Interestingly, five out of six individuals who were temporarily admitted all had collateral involvement (such as contacting caregivers, consulting HCPs outside of the hospital, cross-referencing previous admission notes) recorded in their charts. Individuals who had collateral involvement also had a more detailed history of presenting illness listed in their charts, along with more detailed disposition plans.

## Discussion

This exploratory study is the first to describe STB clinical presentations among autistic adults presenting to a psychiatric ED. An important finding was that of the 24 autistic individuals who presented to the ED over a 10-week period in the larger chart review (see Fernandes et al., 2020), 16 individuals presented to the ED for STB. Among this group, eight individuals presented with STB as their chief complaint, and eight individuals had STB identified by HCPs through secondary screening with a mental status exam. Whilst there likely are many reasons as to why STB was not identified as the presenting issue for 50% of individuals in this study, one contributing factor may be the social-communicative difficulties associated with autism. Unless clearly probed by a HCP, autistic individuals may have particular challenges processing, and verbally expressing their thoughts and feelings about STB in the first place. Our findings suggest that it is imperative to regularly screen for STB when an autistic individual presents to ED. Regularly screening for STB is important as STB has been reported to be the most common psychiatric presentation for autistic adults (Lunsky et al., 2017, Tint et al., 2019) in this particular region where the study was conducted. Screening for STB during ED visits serves as an important opportunity for suicidal risk reduction as autistic children (Kalb et al., 2012) and adults (Vohra et al., 2017) are more likely to visit an ED than non-autistic people.

The complexity in the presentation and impact of STB was compounded by challenges with ruminating and perseverating about STB among some autistic people. Whilst the impact of rumination cannot be ascertained from a chart review, this finding is important as autistic individuals can experience repetitive and ‘sticky thinking’, cognitive inflexibility (Samson et al., 2014), and excessive worrying (Conner & White, 2018). The tendency to perseverate on particular topics such as suicide in this case, can potentially amplify STB risk. This finding is important as rumination is a risk marker for STB in the general population (O’Connor & Kirtely, 2018), and the impact of rumination about STB observed in this study indices an important area of in-depth cognitive and clinical investigation.

In this study, STB commonly occurred during points of life transitions, and was common in emerging adulthood (mean age 22.5 years). This is important, as this subset of individuals tends to be younger than the overall patient population presenting to this ED, which was between 25 and 44 years-old (CAMH internal, 2020). Uncovering the impact of life transition is noteworthy as transition is a risk factor for STB in the general population (Franklin et al, 2017), particularly during emerging adulthood (Thompson & Swartout, 2018). Our findings highlight the need to support the mental health of autistic individuals during this transition period as the onset of major psychiatric challenges typically commence during adolescence and early adulthood for autistic (Lai et al., 2019) and non-autistic people (Pearson et al., 2013). To support autistic adolescents during transition periods, upstream interventions such as coordinated care pathways across education systems, vocational sectors, community support, and healthcare (including the ED) are needed. A coordinated approach currently is lacking in the area where the study was conducted (Hamdani, 2016), and could be a way forward to ensure that autistic people are adequately supported (Jachyra et al., 2019).

In addition to upstream policy interventions with coordinated healthcare pathways, our findings point to the need for immediate support for autistic people in the ED when they are experiencing STB. In addition to regularly screening for STB during ED visits, we suggest four points of departure. First, we encourage autistic people and their families to bring a health information document which provides information about the person’s medical history and potential triggers (see Heifetz & Lunsky, 2018). This information can be critical to provide HCPs with contextual information to guide clinical assessment and dispositions. Second, we encourage HCPs to seek collateral information such as contacting caregivers, support workers, and healthcare professionals (if the autistic individual provides the consent to do so). Engaging with collateral at the height of a crisis may help provide more contextually relevant information and previous life history in an effort to help determine the contributors of distress, which can inform acute care management and disposition (MacKenzie et al., 2013). Third, we encourage HCPs to conduct an exit interview before discharge, as a tool to facilitate communication between HCPs and autistic people about the ED visit. The goal of the exit interview is to review and discuss the ED visit, along with discussing the outcome using clear, simple, and non-technical language (e.g., Malhas et al., 2019). To facilitate comprehension, it may be helpful for HCPs to rephrase and repeat information as needed, and to ask autistic individuals to summarize the ED visit from their perspective to build a shared understanding. It may be helpful to also share the exit interview or discharge summary with caregivers, community workers, and community healthcare professionals (if patient consents) to ensure continuity of care. Fourth, we emphasize the importance of sharing mental health resources developed by autistic people, for autistic people to assist with crisis support and navigation of healthcare services. Resources such as guides for crisis workers working with autistic people (Autistica, 2020), mental health literacy guides (Autism Mental Health Literacy Project Group, 2021); and safety plans to reduce self-harm and suicide for autistic adults (Autism Adapted Safety Plans, 2020) may be of particular utility.

## Limitations

With the majority of our sample being young adults, future work with children, youth and older adults is needed to better understand how autistic individuals experience STB, and how STB manifest in autistic individuals across the lifespan. Future research should also analyze charts of other psychiatric patients or neurodivergent individuals with STB to be able to draw conclusions on the degree of specificity. This study is also limited by the small sample size, and the findings may not be generalizable as the observations are specific to this geographical region and the tertiary psychiatric ED context. Despite these limitations, this exploratory study is a point of departure in advancing knowledge about the qualitative aspects of STB among autistic adults which heretofore has been absent.

## Conclusion

We identified STB presentations in autistic adults in a psychiatric ED setting and uncovered that STB was common, but not always the presenting issue when autistic adults presented to emergency psychiatric care. Explicit efforts are needed to ensure STB amongst autistic people are sufficiently recognized in a timely manner, and adequately understood considering the unique characteristics associated with autism, and related interpersonal and life transition contexts.

## Footnotes

1 In this paper, we use identity-first language (autistic people), as this has been expressed to be the preferred choice in the autistic community. While we use identity-first language in this paper, we recognize the diversity of perspectives and preferences of others who may use person-first language (person with autism).
